# ATAK Complex (Adrenaline, Takotsubo, Anaphylaxis, and Kounis Hypersensitivity-Associated Coronary Syndrome) after COVID-19 Vaccination and Review of the Literature

**DOI:** 10.3390/vaccines11020322

**Published:** 2023-01-31

**Authors:** Paola Lucia Minciullo, Giuliana Amato, Federica Vita, Giovanni Pioggia, Sebastiano Gangemi

**Affiliations:** 1 Unit and School of Allergy and Clinical Immunology, Department of Clinical and Experimental Medicine, University Hospital “G. Martino”, University of Messina, 98125 Messina, Italy; 2 Institute for Biomedical Research and Innovation (IRIB), National Research Council of Italy (CNR), 98164 Messina, Italy

**Keywords:** ATAK, COVID-19 vaccination, adrenaline, Takotsubo cardiomyopathy, anaphylaxis, Kounis hypersensitivity, coronary syndrome

## Abstract

Anaphylactic events triggered by mRNA COVID-19 vaccines are neither serious nor frequent. Kounis syndrome is described as the concomitant occurrence of acute coronary events and hypersensitivity reactions induced by vasospastic mediators after an allergic event. Kounis syndrome caused by vaccines is very rare. Up to now, only a few cases of allergic myocardial infarction after mRNA COVID-19 vaccine administration have been reported. Takotsubo cardiomyopathy is a syndrome characterized by transient wall movement abnormalities of the left ventricular apex, mid-ventricle, or other myocardial distribution, usually triggered by intense emotional or physical stress. Takotsubo cardiomyopathy after COVID-19 vaccine administration has been reported, usually with a delayed onset. A new entity characterized by the association of adrenaline administration, Takotsubo cardiomyopathy, anaphylaxis, and Kounis hypersensitivity was recently described: the ATAK complex. Here, we report a case of Takotsubo cardiomyopathy that occurred together with an anaphylactic reaction to an mRNA COVID-19 vaccine that required the use of adrenaline. The timing of the allergic reaction and the referenced clinical symptoms could not exclude the idea that Kounis syndrome occurred. Therefore, we can assume the patient presented the ATAK complex. We believe that highlighting on this ATAK complex will aid the application of proper diagnostic, preventive and therapeutic measures.

## 1. Introduction

Historically, vaccination seems to be the best prevention strategy to contain infectious diseases. In the COVID-19 pandemic, scientific research focused on the development of vaccines since the beginning: in September 2020, 146 COVID-19 vaccine candidates were in the preclinical phase and 36 vaccine candidates were in the clinical stage [1]. Different vaccine platforms have been employed: nucleic acid mRNA-based vaccines, viral vector vaccines developed with new biotechnology, subunit vaccines, and whole-pathogen inactivated virus vaccines [2]. The mRNA vaccine platform is the most employed during this pandemic. Clinical trials reported that the Pfizer/BioNTech (BNT162b2) mRNA vaccine had a 95% efficacy [3] and the Moderna mRNA vaccine (mRNA1273) had a 94.1% efficacy [4].

Allergic reactions to vaccines are infrequently associated with the active vaccine itself since they might also be induced by an excipient. Excipients are inactive ingredients that act as major agents in the development of specific IgE-mediated and immediate reactions associated with vaccines [5].

Additionally, excipients could cause pseudo0allergic reactions: complement activation-related pseudo-allergy (CARPA), induced by IgM and IgG, represents a nonspecific immune response to PEGylated nanoparticle-based drugs [6].

Excipients in COVID-19 vaccines are heterogeneous. mRNA vaccines such as Pfizer–BioNTech and Moderna contain polyethylene glycol (PEG), and the latter also contains tromethamine [7,8]; viral vector vaccines, such as AstraZeneca, Johnson & Johnson, and Sputnik V, contain polysorbate 80, an excipient that is structurally similar to PEG and is associated with disodium edetate dihydrate (ethylenediaminetetraacetic acid) in the Astra Zeneca vaccine and disodium ethylenediaminetetraacetic acid dehydrate in the Sputnik V vaccine [7]. The inactivated vaccine CoronaVac contains aluminum hydroxide, disodium hydrogen phosphate, sodium dihydrogen phosphate monohydrate, and sodium chloride [9].

These excipients are also found in personal hygiene products such as cosmetics, dental materials, and anticancer drugs, which could sensitize their users [10].

Vaccines may induce allergic reactions through different pathophysiologic mechanisms: IgE-mediated allergic reactions, non-IgE-mediated mast cell degranulation, the Mas-related G protein-coupled receptor X2 (MRGPRX2) activation of mast cells, and type IV hypersensitivity or delayed reactions [5]. IgE-mediated allergic reactions consist of mast cell activation via Fcε receptor-1; they are classical type 1 hypersensitivity reactions and occur within 4 h after antigen exposure [11,12]. Increased levels of serum tryptase and specific IgE detection confirm this mechanism [13]. Serum tryptase can be useful in diagnosis and should be tested at 2 (peak level) and 24 h or later (baseline sample) after the onset of symptoms [14]. The most possible culprits of IgE-mediated reactions are excipients [15].

The C1q, C3a, C4, C5a anaphylatoxins and Factor B may lead to non-IgE-mediated mast cell degranulation. This pathogenetic mechanism involves tryptase and IL-5, and it could lead to renal failure and fatal cerebral events [5,13].

The Mas-related G protein-coupled receptor X2 (MRGPRX2) activation of mast cells could lead to severe allergic reactions [16]. Specific IgEs could be undetectable, and tryptase levels could be normal, even in serious events [15].

Delayed reactions usually appear 48 h after vaccine administration, are independent of antibody and cell mediation, and are induced by cytokine release and the overstimulation of T cells and monocytes/macrophages. Thimerosal and aluminum, for example, could lead to type 4 hypersensitivity reactions [11].

According to a recent study, the risk of hypersensitivity post-vaccination is 1.31 (95% CI, 0.90–1.84) per million vaccine doses [5]. In severe anaphylactic reactions, epinephrine is a first-choice drug [17].

According to recent data from a global meta-analysis, the incidence of anaphylaxis after COVID-19 vaccine administration is estimated to be 7.91 per million vaccine doses (95% confidence interval CI 4.02–15.59), and no reports of deaths from anaphylaxis after COVID-19 vaccination have been described [18].

Usually, anaphylactic events triggered by mRNA COVID-19 vaccines are neither serious nor frequent; most of these cases does not require any treatment [19].

In Italy, according to annual report of the Agenzia Italiana del Farmaco (AIFA), published in February 2022, the rate of anaphylaxis was evaluated to be 3 per million vaccine doses for the Pfizer/Biontech vaccine (Comirnaty) and 1.9 per million vaccine doses for the Moderna vaccine (Spikevax) [20].

During anaphylactic reactions, the massive release of mediators and compensatory catecholamines may cause coronary artery spasms, consequently priming stress-induced cardiomyopathy [5,21].

Anxiety related to vaccine administration due to the fear of developing vaccine side effects has been reported to cause stress-induced cardiovascular damage in response to COVID-19 vaccines [22]. Moreover, tremors, flushing, tachycardia, shortness of breath, inducible laryngeal obstruction throat tightness and vocal cord dysfunction combined with panic and anxiety could appear to be allergic reactions and may complicate proper diagnosis and treatment [5,23]. Vasovagal symptomatology could often occur in individuals, especially in young people and females [24].

In some cases of myocarditis induced by mRNA COVID-19 vaccines, the myocardial biopsy/autopsy showed an inflammatory infiltration of macrophages, T-cells and eosinophils, similar to the features myocarditis hypersensitivity [25].

Additionally, since IgE levels could be increased during acute myocardial infarction and in stable and unstable angina, elevated IgE levels might be correlated with the severity of acute myocardial infarction and weak plaque stability [26] and may be a risk factor for increased cardiovascular mortality [27].

Here, we report a case of a Takotsubo cardiomyopathy that occurred together with an allergic reaction to an mRNA COVID-19 vaccine. The timing of the allergic reaction and the referenced clinical symptoms could not exclude the idea that Kounis syndrome occurred. Since the patient was treated with epinephrine, we could assume that our case was the first case of the ATAK complex, an entity characterized by the association of adrenaline administration, Takotsubo cardiomyopathy, anaphylaxis, and Kounis hypersensitivity after mRNA vaccine administration.

## 2. Case Report

A 54-year-old woman—a social health worker employed in a little hospital—was vaccinated with first dose of the SARS-CoV-2 Pfizer–Biontech vaccine in January 2021 at a hospital vaccination center. Ten minutes after vaccination, she experienced general discomfort, visual changes, and pharyngeal itching. Nevertheless, she left the vaccination center after the observation time (15 min) according to vaccine protocol and returned to her hospital ward. Two hours later, she was carried to the emergency room because of the onset of dyspnea and confusion. Signs of hypovolemia such as tachycardia and hypotension were detected. Therefore, the patient was treated with polygeline iv, hydrocortisone iv, chlorphenamine, methylprednisolone, oxygen therapy, and two consecutive administrations of adrenaline iv (0.5 mg × 1 mL). A few minutes later, the patient developed hematuria, melena, hematemesis, and chest pain.

An ECG revealed ST segment depression (V3–V4), echocardiography revealed lower and lateral akinesia, coronary angiography showed no evidence of coronary artery obstruction/plaque rupture, and cardiac enzymes were all normal.

The patient was transferred to the intensive care unit; echocardiography was repeated, thus confirming lower and lateral akinesia. She was discharged after 4 days with a diagnosis of “Takotsubo syndrome triggered/provoked by allergic shock after SARS-CoV-2 vaccination”.

Therefore, the patient was referred to the Unit of Allergy and Clinical Immunology of the University Hospital of Messina. Hypothesizing PEG sensitization, the patient underwent to skin tests for PEG according to the guidelines of the Italian Society of Allergy, Asthma and Clinical Immunology. The test showed that she did not have nonspecific skin hyper-reactivity. Therefore, the patient was asked to perform a basophil activation test for PEG, which was negative. Her serum tryptase level was 9.8 ug/L (normal range < 11), and her total IgE level was normal (37, 7 UI/mL). However, the patient underwent coloscopy one year prior to her vaccination without reactions, for which she prepared with PEG, and used PEG-containing cosmetics in the weeks before the event.

The patient was considered clinically unsuitable to receive a SARS-CoV-2 vaccine, and she obtained a permanent medical exemption.

Subsequently, the woman was twice infected with COVID-19 and recovered without complications.

The patient did not take drugs in chronic therapy for any pathological condition, but her medical history showed previous adverse reactions to other drugs: widespread erythema after intravenous iron therapy and conjunctival hyperemia and the sensation of tongue swelling after the use of acetylsalicylic acid. In both events, symptoms regressed after systemic corticosteroid therapy.

## 3. Discussion

Recently, adrenaline administration, Takotsubo, anaphylaxis, and Kounis hypersensitivity-associated coronary syndrome (ATAK) was established as a challenging contemporary complex representing a possible overlap between Takotsubo cardiomyopathy and Kounis syndrome.

**Figure 1:**
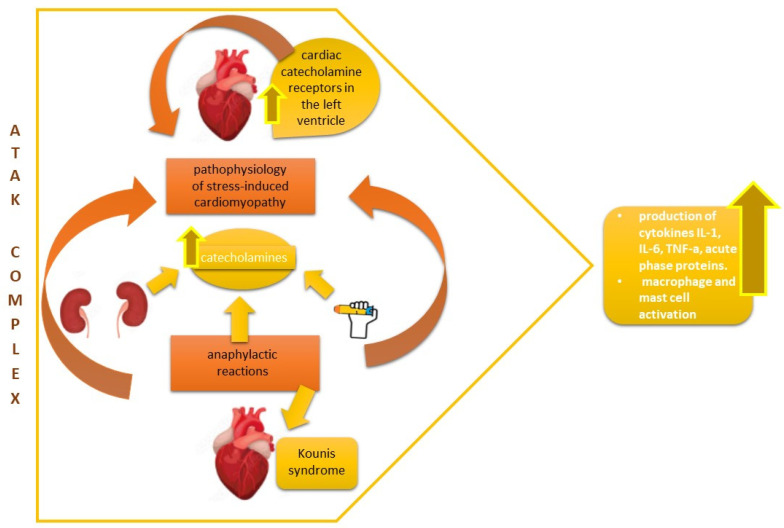
ATAK complex.

Kounis syndrome (KS) is described as the coincidental occurrence of acute coronary events and hypersensitivity reactions induced by vasospastic mediators after an allergic event caused by food ingestion or drug administration combined with mast cell and platelet activation [28]. Clinical studies have indicated that various simultaneously administered drugs could simulate the action of potential antigens through an additive effect, thus inducing anaphylactic cardiogenic shock and triggering cells to release their mediators with the danger of perpetuating this vicious cycle [29] (see Figure 1).

Kounis syndrome could occur through various mechanisms and is classified as four different types. Type I is characterized by a coronary spasm in normal coronary arteries, type II is described as a coronary spasm or an atheromatous break plaque with a preexisting atheromatous disease, and type III is defined a stent thrombosis. Recently, Giovannini et al. established type IV, which involves a coronary artery bypass graft thrombosis [30].

In clinical practice, KS risks being underestimated. KS is more frequent in men and middle-aged people: non-steroidal anti-inflammatory drugs, antibiotics, other drugs and hymenoptera venom have been reported as potential trigger factors. Cardiogenic shock can hide skin manifestations of anaphylaxis by inhibiting or delaying released mediators that could elicit itching or rashes. KS could also be considered in the absence of systemic hypersensitivity manifestations; indeed, respiratory symptoms have been reported in only 15% of cases. Type I is the most common (72.6%), and type III is the least frequent (5.1%) [14].

Although vaccine components may be regarded as possible agents of allergic reactions, KS caused by vaccines is a very rare medical issue [5]. Myocardial infarction after COVID-19 vaccine administration has been associated with Kounis hypersensitivity in only a few cases, probably because of the frequent absence of a history of allergies [31,32,33]. Different types of COVID-19 vaccines and their excipients (mainly polysorbate 80 and PEG) have been involved [34].

Potential allergic reactions that also could be classified as KS, characterized by myocardial infarction, have been reported with different types of vaccines. One case of a STEMI was found in a healthy 63-year-old man two days after the administration of the AstraZeneca vaccine. Typical central chest pain was reported approximately 1.5 h after receiving the first dose of an inactive COVID-19 vaccine in one case [34] and approximately one hour after receiving a healthy 96-year-old female received her first dose of the Moderna vaccine in another case [35].

These cases do not mean that the COVID-19 vaccine has been directly causing myocardial infarctions, since this cardiomyopathy is a frequent diagnosis in daily practice and mass vaccination against COVID-19 took place; therefore, these cases could have been coincidences, or perhaps vaccines can induce an overload in the heart, acting as predisposing factor. In these cases, KS could be a potential mechanism that clarifies the link between vaccines and myocardial infarction [5].

Takotsubo cardiomyopathy is typically characterized by transient wall movement abnormalities of the left ventricular apex, mid-ventricle, or other myocardial distributions. It is usually triggered by intense emotional or physical stresses, and it above-all affects postmenopausal women due to estrogen deficiency [36]. This stress-induced cardiomyopathy has been excessively debated. Although KS, pheochromocytoma crisis, mastocytosis, hymenoptera stings, several drugs, and vaccines, among other factors, have been implicated in Takotsubo cardiomyopathy, many questions regarding its etiology, pathophysiology, and treatment remain unanswered [37].

Cardiac complications associated with COVID-19 mRNA vaccines are rare (apart from an inflammatory state such as pericarditis and myocarditis), and some cases of Takotsubo cardiomyopathy have been reported [38]. In most cases, symptom onset after vaccination was delayed [38,39,40,41,42]; the exceptions were two cases after the first dose [43,44] and one case after the second dose [45] (see Table 1). Anaphylactic reactions and/or adrenaline treatment have not been reported.

Additionally, Takotsubo cardiomyopathy has been detected not only after anaphylaxis but also in circumstances that require treatment for anaphylaxis. Histamine and catecholamine increases play pivotal roles in the pathophysiology of stress-induced cardiomyopathy because they can induce an excessive activation of cardiac catecholamine receptors in the left ventricle. Moreover, the possible injection of catecholamines, such as norepinephrine or epinephrine, for hemodynamic depletion support could also increase the plasma catecholamine level and worsen medical outcomes [21].

In the literature, only four reports linking Takotsubo cardiomyopathy and Kounis syndrome by adrenergic endogenous and exogenous release, thus supporting the ATAK hypothesis, have been described. These cases occurred in different conditions. In 2018, a 60-year-old female with a history of smoking, depression, allergy to aspirin, hypothyroidism, and systemic arterial hypertension developed anaphylactic shock during plasma expander (gelofusine) administration. When the sinus rhythm stabilized, the patient was subjected to an electrocardiogram that showed mild ST elevation in the anterior precordial leads and T wave changes; moreover, alterations of left ventricular apex kinesis were seen in echocardiography and normal coronary arteries were detected with coronary angiography [48]. In 2022, three patients experienced anaphylactic events after blood component transfusion [49]; in 2019, a 60-year-old female with a history of insulin-dependent diabetes mellitus, hypertension, chronic kidney disease, and multiple myeloma (MM) presented chest pain and dyspnea during treatment with anti-CD38 antibodies (daratumumab). A sinus rhythm with biphasic T waves in V2 and V3, along with deep T wave inversions in V4 through V6 (suggestive of Wellens syndrome) was detected with an ECG, and apical and anteroseptal akinesis were revealed by an echocardiogram (a previous echocardiogram performed 3 months before was normal). Normal coronary arteries were revealed by emergency cardiac catheterization [50]. In 2015, Gicquel-Schlemmer et al. reported a case of fatal Takotsubo cardiomyopathy in a 48-year-old female during shoulder arthroscopy; the patient was in treatment with beta-blockers for hypertension. A previous cardiology workup (about a year before) and the clinical examination during the presurgical anesthesiology consultation were normal [51]. In 2016, Kounis et al. identified the ATAK complex in this case of fatal Takotsubo cardiomyopathy due to epinephrine hypersensitivity and worsened by the beta-blocker therapy [29].

In patients with KS, complete blood count, cardiac biomarkers, d-dimer, NT-proBNP, serum tryptase, and eosinophil levels could be essential for the identification of this syndrome; some nonspecific ST-T wave changes, ST segment depression, or elevation may be observed; otherwise, the ECG could be absolutely normal [32].

During an anaphylactic event, reduced venous return, systemic vasodilation and volume loss due to increased vascular permeability may not be the leading causes for hemodynamic disorders. Indeed, studies have reported many cases in which hemodynamic status was unresponsive to the intravenous administration of fluids because of myocardial stunning and ventricular hypokinesia mimicking Takotsubo cardiomyopathy [52].

In our case, it could be hypothesized that the initial allergic reaction after vaccination and the consecutive epinephrine administration evolved towards Takotsubo cardiomyopathy and KS overlap, which can be described as the ATAK complex. To our knowledge, this report represents the first of such a case after COVID-19 vaccination. Although the association does not describe real causality, this case may increase awareness of a possible correlation between COVID-19 vaccines and the ATAK complex.

## 4. Conclusions

Vaccines constitute the most effective weapons against viral diseases. However, allergic reactions may happen during vaccination. Physicians should be conscious that KS induced by a coronavirus vaccine is a rare but relevant reaction. Anyone who develops post-vaccination chest pain or anaphylaxis should be examined via ECG, echocardiography, and cardiac markers, and they should be kept under observation for an adequate period or hospitalized if necessary.

We therefore believe that highlighting the ATAK complex could aid the application of proper diagnostic, preventive, and therapeutic measures.

## Figures and Tables

**Table 1:** Takotsubo cardiomyopathy and Kounis syndrome reports after COVID-19 vaccine administration.

Reference	Age	Sex	Diagnosis	mRNA Vaccine Dose	Time from Vaccination to Symptom	Symptoms	Cardiac Test	Treatment
Year								
Authors								
[39]	63 Y	female	Takotsubo cardiomyopathy	1	1 day	Fever and dyspnea.	ECG: Negative T waves over the inferior/anterior leads. Angiography: normal.	N/A
2021								
Berto et al.								
[40]	60 Y	female	Takotsubo cardiomyopathy	2	4 days	Chest pain.	ECG: Inferolateral T wave inversions. Angiography: normal.	Metoprolol and Lisinopril
2021								
Vidula et al								
[41]	73 Y	male	Takotsubo cardiomyopathy	2	17 h	Dyspnea, fatigue, chest pain, shortness of breath, and orthopnea.	ECG: ST changes in inferolateral leads, poor anterior R wave progression. Angiograph: normal.	Furosemide IV diuresis, metoprolol, and Losartan
2021								
Fearon et al.								
[42]	65 Y	female	Takotsubo cardiomyopathy	1	1 day	Chest pain, myalgia, nausea, and headache.	ECG: abnormal	Aspirin, atorvastatin, lisinopril, and metoprolol succinate
2021								
Jani et al.								
[43]	44 Y	female	Takotsubo cardiomyopathy	1	15 min	Chest pain palpitation.	ECG: ST elevations in the inferolateral leads. Angiograph: normal.	Conservative treatment
2021								
Lee et al.								
[44]	71 Y	female	Takotsubo cardiomyopathy	1	5 h	Chest pain and shortness of breath.	ECG: abnormal.	N/A
2022								
Tedeschi et al.								
[31]	64 Y	male	Kounis III	1	immediately	Chills, chest pain, pallor, diaphoresis, and hypotension.	ECG: ST segment elevation in the anteroseptal precordial leads. Angiography: stent thrombosis in the proximal segment of the left anterior descending artery and TIMI grade 0 flow.	N/A
2022								
Chadi Allam et al.								
[46]	59 Y	male	Kounis III	1	20 min	Precordial pain, sweat, and discrete micropapular rash on chest. No exanthema, pruritus, dyspnea, wheezing, diarrhea, or abdominal pain.	ECG: showed sinus rhythm, pathological Q waves and T wave inversion in V2–V5 leads and ST segment elevation in II, III, and aVF leads. An ST elevation myocardial infarction (STEMI) was admitted. Angiography: evidence of stent thrombosis of right coronary artery.	Clopidogrel and Rivaroxaban
2022								
Fihalo et al.								
[32]	41 Y	female	Kounis I	1 (CoronaVac)	15 min	Flushing, palpitation, lip and tongue swelling, shortness of breath, and chest pain.	ECG: poor R wave progression in precordial leads, V4–6 T wave inversion, and fragmented QRS in aVL. Angiography: no sign of coronary atherosclerosis.	Aspirin, oral antihistamines, diltiazem, and corticosteroid
2021								
Ozdemir et al.								
[47]	22 Y	female	Kounis I	1	15 min	On admission, vital signs were stable besides a mild tachycardia; during follow-up, the patient had increased complaints including shortness of breath and chest pain.	ECG: ST segment elevations in the inferior and anterior derivations (D2, D3, avF, and V3–6). Angiography: no abnormalities.	Acetyl salicylic acid (300 mg), pheniramine maleate (45.5 Mg), and dexamethasone (8 mg)
2022								
Şancı E. et al								

Abbreviation: N/A: not available; Y: Years old.

## Data Availability

Not applicable.

## References

[1] Dong Y., Dai T., Wei Y., Zhang L., Zheng M., Zhou F. (2020). A systematic review of SARS-CoV-2 vaccine candidates. Signal Transduct. Target. Ther..

[2] Pormohammad A., Zarei M., Ghorbani S., Mohammadi M., Razizadeh M.H., Turner D.L., Turner R.J. (2021). Efficacy and Safety of COVID-19 Vaccines: A Systematic Review and Meta-Analysis of Randomized Clinical Trials. Vaccines.

[3] Polack F.P., Thomas S.J., Kitchin N., Absalon J., Gurtman A., Lockhart S., Perez J.L., Pérez Marc G., Moreira E.D., Zerbini C. (2020). Safety and Efficacy of the BNT162b2 mRNA Covid-19 Vaccine. N. Engl. J. Med..

[4] Baden L.R., El Sahly H.M., Essink B., Kotloff K., Frey S., Novak R., Diemert D., Spector S.A., Rouphael N., Creech C.B. (2021). Efficacy and Safety of the mRNA-1273 SARS-CoV-2 Vaccine. N. Engl. J. Med..

[5] Kounis N.G., Koniari I., de Gregorio C., Velissaris D., Petalas K., Brinia A., Assimakopoulos S.F., Gogos C., Kouni S.N., Kounis G.N. (2021). Allergic reactions to current available covid-19 vaccinations: Pathophysiology, causality, and therapeutic considerations. Vaccines.

[6] Neun B.W., Barenholz Y., Szebeni J., Dobrovolskaia M.A. (2018). Understanding the Role of Anti-PEG Antibodies in the Complement Activation by Doxil in Vitro. Molecules.

[7] Haq H.N., Khan H., Chaudhry H., Nimmala S., Demidovich J., Papudesi B.N., Potluri S.D. (2022). Pfizer-BioNTech (BNT162b2), Moderna (mRNA-1273) COVID-19 mRNA vaccines and hypersensitivity reactions. J. Natl. Med. Assoc..

[8] Rutkowski K., Mirakian R., Till S., Rutkowski R., Wagner A. (2021). Adverse reactions to COVID-19 vaccines: A practical approach. Clin. Exp. Allergy J. Br. Soc. Allergy Clin. Immunol..

[9] Laisuan W., Wongsa C., Chiewchalermsri C., Thongngarm T., Rerkpattanapipat T., Iamrahong P., Ruangwattanachok C., Nanthapisal S., Sompornrattanaphan M. (2021). CoronaVac COVID-19 Vaccine-Induced Anaphylaxis: Clinical Characteristics and Revaccination Outcomes. J. Asthma Allergy.

[10] Lyapina M.G., Stoyanova Dencheva M. (2019). Contact sensitization to ingredients of dental materials and cosmetics in dental students: A pilot study. Cent. Eur. J. Public Health.

[11] Chung E.H. (2014). Vaccine allergies. Clin. Exp. Vaccine Res..

[12] Olivera Mesa D., Hogan A.B., Watson O.J., Charles G.D., Hauck K., Ghani A.C., Winskill P. (2022). Modelling the impact of vaccine hesitancy in prolonging the need for Non-Pharmaceutical Interventions to control the COVID-19 pandemic. Commun. Med..

[13] Khan S. (2020). Mast cell tryptase level should be checked in all patients with suspected Kounis syndrome. Eur. Heart J..

[14] Abdelghany M., Subedi R., Shah S., Kozman H. (2017). Kounis syndrome: A review article on epidemiology, diagnostic findings, management and complications of allergic acute coronary syndrome. Int. J. Cardiol..

[15] Stone C.A.J., Rukasin C.R.F., Beachkofsky T.M., Phillips E.J. (2019). Immune-mediated adverse reactions to vaccines. Br. J. Clin. Pharmacol..

[16] Porebski G., Kwiecien K., Pawica M., Kwitniewski M. (2018). Mas-Related G Protein-Coupled Receptor-X2 (MRGPRX2) in Drug Hypersensitivity Reactions. Front. Immunol..

[17] Roh E.J., Lee M.H., Song K.B., Lee Y.K., Kim M.K., Kim T.E., Chung E.H. (2020). Vaccine-related anaphylaxis cases confirmed by KCDC from 2001–2016. J. Korean Med. Sci..

[18] Greenhawt M., Abrams E.M., Shaker M., Chu D.K., Khan D., Akin C., Alqurashi W., Arkwright P., Baldwin J.L., Ben-Shoshan M. (2021). The Risk of Allergic Reaction to SARS-CoV-2 Vaccines and Recommended Evaluation and Management: A Systematic Review, Meta-Analysis, GRADE Assessment, and International Consensus Approach. J. Allergy Clin. Immunol. Pract..

[19] Shimabukuro T., Nair N. (2021). Allergic Reactions including Anaphylaxis after Receipt of the First Dose of Pfizer-BioNTech COVID-19 Vaccine. JAMA-J. Am. Med. Assoc..

[20] del Farmaco A.I. (2021). Rapporto Annuale Sulla Sicurezza dei Vaccini Anti-COVID-19.

[21] Cheng T.O., Kounis N.G. (2012). Takotsubo cardiomyopathy, mental stress and the Kounis syndrome. Int. J. Cardiol..

[22] Sakamoto T., Kagawa Y., Endo A., Tanabe K. (2021). Intense Emotional Stress Over Potential Coronavirus Disease Vaccination Side Effects Leads to Takotsubo Cardiomyopathy. Circ. Rep..

[23] Banerji A., Wickner P.G., Saff R., Stone C.A.J., Robinson L.B., Long A.A., Wolfson A.R., Williams P., Khan D.A., Phillips E. (2021). mRNA Vaccines to Prevent COVID-19 Disease and Reported Allergic Reactions: Current Evidence and Suggested Approach. J. Allergy Clin. Immunol. Pract..

[24] McMurtry C.M. (2020). Managing immunization stress-related response: A contributor to sustaining trust in vaccines. Can. Commun. Dis. Rep..

[25] Kounis N.G., Koniari I., Mplani V., Kouni S., Velissaris D., Plotas P., Tsigkas G. (2022). Rare Hypersensitivity Myocardial Reactions Following COVID-19 Vaccination: Hypersensitivity Myocardial Infarction (Kounis Syndrome) and Hypersensitivity Myocarditis. Anatol. J. Cardiol..

[26] Kounis N.G., Hahalis G. (2016). Serum IgE levels in coronary artery disease. Atherosclerosis.

[27] Guo X., Yuan S., Liu Y., Zeng Y., Xie H., Liu Z., Zhang S., Fang Q., Wang J., Shen Z. (2016). Serum IgE levels are associated with coronary artery disease severity. Atherosclerosis.

[28] Kounis N.G., Zavras G.M. (1991). Histamine-induced coronary artery spasm: The concept of allergic angina. Br. J. Clin. Pract..

[29] Kounis N.G., Soufras G.D. (2016). Shoulder arthroscopy and ATAK (adrenaline, Takotsubo, anaphylaxis, and Kounis hypersensitivty-associated syndrome). Orthop. Traumatol. Surg. Res..

[30] Giovannini M., Koniari I., Mori F., Barni S., Novembre E., Kounis N.G. (2021). Kounis syndrome: Towards a new classification. Int. J. Cardiol..

[31] Allam C., Kounis N.G., Chlawit R., Saouma M., Badaoui G. (2022). Kounis syndrome following COVID-19 vaccination. Baylor University Medical Center Proceedings.

[32] Özdemir İ.H., Özlek B., Özen M.B., Gündüz R., Bayturan Ö. (2021). Type 1 Kounis syndrome induced by inactivated SARS-COV-2 vaccine. J. Emerg. Med..

[33] Tajstra M., Jaroszewicz J., Gąsior M. (2021). Acute coronary tree thrombosis after vaccination for COVID-19. Cardiovasc. Interv..

[34] Maadarani O., Bitar Z., Elzoueiry M., Nader M., Abdelfatah M., Zaalouk T., Mohsen M., Elhabibi M. (2021). Myocardial infarction post COVID-19 vaccine—Coincidence, Kounis syndrome or other explanation—Time will tell. JRSM Open.

[35] Boivin Z., Martin J. (2021). Untimely Myocardial Infarction or COVID-19 Vaccine Side Effect. Cureus.

[36] Amin H.Z., Amin L.Z., Pradipta A. (2020). Takotsubo Cardiomyopathy: A Brief Review. J. Med. Life.

[37] Koniari I., Tzanis G., Tsigkas G., Soufras G., Hahalis G., Kounis N. (2017). Attacking the ATAK Complex in Cardiac Anesthesia. J. Cardiothorac. Vasc. Anesth..

[38] Fazlollahi A., Zahmatyar M., Noori M., Nejadghaderi S.A., Sullman M.J.M., Shekarriz-Foumani R., Kolahi A.A., Singh K., Safiri S. (2021). Cardiac complications following mRNA COVID-19 vaccines: A systematic review of case reports and case series. Rev. Med. Virol..

[39] Berto M.B., Spano G., Wagner B., Bernhard B., Häner J., Huber A.T., Gräni C. (2021). Takotsubo cardiomyopathy after mRNA COVID-19 vaccination. Heart Lung Circ..

[40] Vidula M.K., Ambrose M., Glassberg H., Chokshi N., Chen T., Ferrari V.A., Han Y. (2021). Myocarditis and other cardiovascular complications of the mRNA-based COVID-19 vaccines. Cureus.

[41] Fearon C., Parwani P., Gow-Lee B., Abramov D. (2021). Takotsubo syndrome after receiving the COVID-19 vaccine. J. Cardiol. Cases.

[42] Jani C., Leavitt J., Al Omari O., Dimaso A., Pond K., Gannon S., Chandran A.K., Dennis C., Colgrove R. (2021). COVID-19 Vaccine-Associated Takotsubo Cardiomyopathy. Am. J. Ther..

[43] Lee E., Chew N.W., Ng P., Yeo T.J. (2021). A spectrum of cardiac manifestations post Pfizer-BioNTech COVID-19 vaccination. QJM.

[44] Tedeschi A., Camilli M., Ianni U., Tavecchia G., Palazzini M., Cartella I., Gentile P., Quattrocchi G., Maria Spanò F., Cipriani M. (2022). Takotsubo syndrome after BNT162b2 mRNA COVID-19 vaccine: Emotional or causative relationship with vaccination?. Int. J. Cardiol. Heart Vasc..

[45] Khalid Ahmed S., Gamal Mohamed M., Abdulrahman Essa R., Abdelaziz Ahmed Rashad Dabou E., Omar Abdulqadir S., Muhammad Omar R. (2022). Global reports of takotsubo (stress) cardiomyopathy following COVID-19 vaccination: A systematic review and meta-analysis. IJC Heart Vasc..

[46] Fialho I., Mateus C., Martins-dos-Santos G., Pita J., Cabanelas N., Baptista S.B., Roque D. (2022). Recurrent Kounis syndrome—A life-threatening event after COVID-19 vaccine administration. J. Cardiol. Cases.

[47] Şancı E., Örçen C., Çelik O.M., Özen M.T., Bozyel S. (2022). Kounis syndrome associated with BNT162b2 mRNA COVID-19 vaccine presenting as ST-elevation acute myocardial infarction. Anatol. J. Cardiol..

[48] Margonato D., Abete R., Di Giovine G., Delfino P., Grillo M., Mazzetti S., Poggio D., Rossi J., Khouri T., Mortara A. (2019). Takotsubo cardiomyopathy associated with Kounis syndrome: A clinical case of the “ATAK complex”. J. Cardiol. Cases.

[49] Badami K.G. (2022). Transfusion double whammy? Adrenaline-takotsubo-anaphylaxis-Kounis complex post transfusion?. Vox Sang..

[50] Mustehsan M.H., Jahufar F., Arora S. (2019). A Diagnostically Challenging Infusion Reaction-Kounis, Takotsubo, or the ATAK!. JAMA Intern. Med..

[51] Gicquel-Schlemmer B., Beller J.-P., Mchalwat A., Gicquel P. (2015). Fatal Takotsubo cardiomyopathy due to epinephrine in shoulder arthroscopy. Orthop. Traumatol. Surg. Res..

[52] Soufras G.D., Kounis N.G. (2013). Adrenaline administration for anaphylaxis and the risk of takotsubo and Kounis syndrome. Int. J. Cardiol..

